# A Novel Cyclophilin B Gene in the Red Tide Dinoflagellate* Cochlodinium polykrikoides*: Molecular Characterizations and Transcriptional Responses to Environmental Stresses

**DOI:** 10.1155/2017/4101580

**Published:** 2017-10-26

**Authors:** Sofia Abassi, Hui Wang, Bum Soo Park, Jong-Woo Park, Jang-Seu Ki

**Affiliations:** ^1^Department of Biotechnology, Sangmyung University, Seoul 03016, Republic of Korea; ^2^Institute of Natural Sciences, Sangmyung University, Seoul 03016, Republic of Korea; ^3^Ocean Climate and Ecology Research Division, National Institute of Fisheries Science, Busan 46083, Republic of Korea

## Abstract

The marine dinoflagellate* Cochlodinium polykrikoides* is one of the most common ichthyotoxic species that causes harmful algal blooms (HABs), which leads to ecological damage and huge economic loss in aquaculture industries. Cyclophilins (CYPs) belong to the immunophilin superfamily, and they may play a role in the survival mechanisms of the dinoflagellate in stress environments. In the present study, we identified a novel cyclophilin gene from* C. polykrikoides* and examined physiological and gene transcriptional responses to biocides copper sulphate (CuSO_4_) and sodium hypochlorite (NaOCl). The full length of* CpCYP* was 903 bp, ranging from the dinoflagellate splice leader (DinoSL) sequence to the polyA tail, comprising a 639 bp ORF, a 117 bp 5′-UTR, and a 147 bp 3′-UTR. Motif and phylogenetic comparisons showed that CpCYP was affiliated to group B of CYP. In biocide stressors, cell counts, chlorophyll *a*, and photosynthetic efficiency (*Fv*/*Fm*) of* C. polykrikoides* were considerably decreased in both exposure time- and dose-dependent manners. In addition,* CpCYP* gene expression was significantly induced after 24 h exposure to the biocide-treated stress conditions. These results indicate an effect of the biocides on the cell physiology and expression profile of* CpCYP*, suggesting that the gene may play a role in environmental stress responses.

## 1. Introduction

Dinoflagellate algae are eukaryotic protists which exhibit a great diversity of form and are the most important primary producer in aquatic environments. However, some species (e.g.,* Alexandrium tamarense, Akashiwo sanguinea, *and* Cochlodinium polykrikoides*) can grow very fast when environmental conditions are appropriate, leading to the formation of harmful algal blooms (HABs). These events cause serious environmental damage on fisheries and aquatic ecosystems [1]. The dinoflagellate* C. polykrikoides* is one of the notorious HAB species, and it has expanded oceanic distributions worldwide [2, 3]. In addition, red tides caused by this species can produce ichthyotoxins causing deleterious impacts on the marine ecosystem and aquaculture industries and lead consequently to huge economic losses [4–6].

In molecular aspects, dinoflagellates have extraordinary genomic features, such as large nuclear genome size, fewer histones, permanently condensed and liquid-crystalline chromosomes, ~70% replacement of thymine with 5-hydroxymethyluracil, and extensively methylated nucleotides [7, 8]. Even their genes are expressed to be matured in a* trans*-splicing manner in mRNA processing reactions [9], and some are posttranscriptionally or translationally regulated [10]. For these reasons, they have been widely used in studies related to gene and genome researches, protist molecular evolution, and even recent ecotoxicological assessments [11–15].

Cyclophilins (CYPs) are a subgroup of a large family of proteins called immunophilins, with a peptidyl-prolyl* cis-trans* isomerase (PPIase) activity [16]. PPIases catalyze the* cis*-*trans* isomerization of proline imidic peptide bonds and regulate protein folding and maturation. They are found in a large variety of organisms and thus are highly conserved. All CYPs share a common domain of approximately 109 amino acids, the cyclophilin-like domain (CLD), surrounded by domains unique to each member of the family that is associated with subcellular compartmentalization and functional specialization [17]. CYPs can be found in most cellular compartments of most tissues and encode unique functions. They also have varying degrees of affinity for the immunosuppressive drug CsA, a cyclic 11-amino-acid peptide produced by the fungus* Tolypocladium inflatum*. Cyclophilin A (CYP A), in particular, is the major intracellular receptor for CsA [18]. The 18 kDa archetypal CYP A is cytosolic and is found in all tissues in mammals, whereas other cyclophilins, whether they have a CLD alone or in combination with other domains, are found in the endoplasmic reticulum (ER), the mitochondria, or the nucleus. In mammals, CYP A and CYP 40 are cytosolic, whereas other groups, CYP B and CYP C, have amino-terminal signal sequences that target them to the ER protein secretory pathway [16, 19]. These findings show that CYPs are divided into groups (CYP A, CYP B, CYP C, etc.) based on their size and their target location [20], and thus they may have different roles rather than already-known housekeeping functions.

Recent studies have shown that CYPs belong to different subcellular compartments, and they have been found to have diverse roles, including detoxification of reactive oxygen species (ROS), protein maturation processing, immune response, gene regulation by microRNA activity, and spliceosome assembly [21]. Besides the PPIase and protein chaperone activities (housekeeping functions), CYPs function in mitochondrial metabolism, apoptosis, immunological response, inflammation, and cell growth and proliferation [22–24]. Regarding algae, Wu et al. [25] studied the expression of some genes in the green seaweed* Ulva fasciata* and revealed that CYP was involved in redox homeostasis and antioxidant defense. In addition, the role of CYP in response to stress was previously reported from the red algae* Porphyra haitanensis* [26]. Moreover, the CYPs could play a critical role in the regulation of cnidarian-algal symbiosis [27]. Perez and Weis [28] suggested that CYP may help in the regulation of symbiosis between the sea anemone* Aiptasia pallida* and intracellular dinoflagellates. Interestingly, their study showed that the dinoflagellate symbionts (zooxanthellae) became heat sensitive when CYP was inhibited by cyclosporine and showed loss of the symbionts from the host tissues [28]. More recently, we found that the dinoflagellate* Prorocentrum minimum* has stress responsive functions in cells exposed to heavy metals and organic contaminants [29]. These findings suggest that dinoflagellate CYPs may be considered as a sensitive indicator for environmental contaminants; however, their stress responsive involvement is not tested widely by using other dinoflagellates. In addition, different types of CYPs (e.g., CYP A, CYP B, CYP C, and CYP D) have not been characterized by comparison to those of other eukaryotes to date. Hence, discovering more information about CYP gene structure and expressional responses in other species shall be appropriate and informative, which is beneficial to understand the gene regulation mechanisms in adaptive, survival strategies of dinoflagellates.

In the present study, we determined the full sequence of a type CYP gene from the dinoflagellate* Cochlodinium polykrikoides (CpCYP)*. We performed a series of analyses to characterize* CpCYP* gene and genomic features, including motif searches, intergenic spacer (IGS), deduced protein sequence comparisons, and phylogenetic relationships. In addition, we examined the transcriptional response of* CpCYP* under stress conditions using two biocides: CuSO_4_ and NaOCl.

## 2. Materials and Methods

### 2.1. Cell Culture

The strain (Cp-01) of* C. polykrikoides *was obtained from the National Institute of Fisheries Science (Busan, Korea). The cell cultures were maintained in f/2 medium at 20°C in a 12 : 12 h light : dark cycle with a photon flux density of approximately 65 *μ*mol photons m^−2^ s^−1^.

### 2.2. RNA Extraction, cDNA Synthesis, and DNA Extraction

*C. polykrikoides* cultures were harvested by centrifugation at 3500 rpm for 5 min, frozen immediately in liquid nitrogen, and stored at −80°C until RNA extraction. The cells were physically broken by freeze-thawing in liquid nitrogen and homogenized by Mini-Bead beater (BioSpec Products Inc., Bartlesville, OK) with zirconium beads (diameter: 0.1 mm). Total RNA was isolated using TRIzol (Invitrogen, Carlsbad, CA) and purified by Mini Spin Columns of RNeasy Mini Kit (Qiagen, Valencia, CA).

For the first-strand cDNA, 2 different cDNA synthesis kits were employed: TOPscript™ cDNA Synthesis Kit for the gene cloning of* CpCYP* and TOPscript RT DryMIX (dN6 plus) for gene expression study. Then, the first-strand cDNA templates were diluted 1 : 10 with nuclease-free water for use in subsequent analyses. Total genomic DNA was extracted from* C. polykrikoides *following the cetyltrimethylammonium bromide (CTAB) method [30].

### 2.3. Determination of CYP Gene Sequences

The full length of* CpCYP* sequence was determined by rapid amplification of cDNA ends (RACE). Partial gene sequences of* CpCYP *were taken from an expressed sequence tags (ESTs) database of* C. polykrikoides* and were used to design the primers for the full-length amplification of* CpCYP *(Table 1). The 3′- and 5′-untranslated regions (UTRs) of* CpCYP* were determined by using the 3′- and 5′-RACE, respectively. For the RACE, the primary and secondary PCRs conditions were as follows: predenaturation at 96°C for 10 min; 35 cycles of 95°C for 30 s, 55°C/58°C for 30 s, and 72°C for 80 s; and extension at 72°C for 10 min, respectively. Positive core PCR products were purified, cloned into pTOP TA V2 vector (Enzynomics, Daejeon, Korea), transformed into* E. coli* competent cells, and subjected to sequencing.* CpCYP *full-length sequence was validated by PCR with specific primers (Table 1). As for the determination of* CpCYP* genomic sequence, the used primers were designed according to cDNA sequence (Table 1).

### 2.4. *CpCYP* Characterization and Phylogenetic Analyses

The similarities of* CpCYP* aa sequences with those of other species were assessed by using BioEdit 7.0.5.3 [31]. The signal peptide prediction, proteins motifs, and conserved domain of* CpCYP* protein were analyzed using online servers and databases, including InterPro 62.0 (http://www.ebi.ac.uk/interpro/), SignalP 4.1 (http://www.cbs.dtu.dk/services/SignalP/), PROSITE (http://prosite.expasy.org/), Compute pI/Mw tool (http://web.expasy.org/compute_pi/), and NCBI Conserved Domain Database (https://www.ncbi.nlm.nih.gov/Structure/cdd/wrpsb.cgi).

Phylogenetic analysis of CpCYP and other CYPs was performed in MEGA5 [32], using the neighbor-joining (NJ) method [33]. A bootstrap consensus tree inferred from 1,000 replicates was adopted to represent the evolutionary history of the taxa analyzed [34]. The tree was drawn to scale, with branch lengths in the same units as those of the evolutionary distances used to infer the phylogenetic tree. The evolutionary distances were computed using the JTT matrix-based method [35] and were in the units of the number of amino acid substitutions per site.

### 2.5. Toxicant Treatments and Photosynthesis Efficiency

Exponential phase cells were used for toxicant treatments. Typical toxicants and biocides CuSO_4_ (cat. number C1297, Sigma, MO) and NaOCl (cat. number 425044, Sigma, MO) were employed in the present study.

The maximal photosynthesis efficiency (*Fv*/*Fm*) was assessed for different doses of toxicants. Before measuring the fluorescence for* Fv*/*Fm*, all the samples were allowed to adapt in the dark for 25 min. The fluorescence efficiency rates were measured using a Handy Plant Efficiency Analyser fluorometer (Handy PEA fluorimeter; Hansatech Instruments Ltd., King's Lynn, UK). The basic fluorescence parameters [36] are as follows:

 
*Fo*: minimal fluorescence in the dark-adapted state
 
*Fm*: maximal fluorescence in the dark-adapted state
 
*Fv*: variation in fluorescence
 
*Fv/Fm* = (*Fm* − *Fo*/*Fm*): maximal quantum yield of PS II photochemistry

### 2.6. Gene Expression and Statistical Analysis

The dose effects of toxicants (CuSO_4_ and NaOCl) on* CpCYP* transcriptional expression were tested using* C. polykrikoides* cultures treated with a series of concentrations of each toxicant. Different concentrations (0.05, 0.1, 0.2, 0.5, and 1.0 mgL^−1^) were chosen considering the EC_50_ values of the biocides [37]. The treated and untreated cultures were harvested at 24 h for the analysis. RNA extraction and cDNA synthesis were described above. All quantitative real-time polymerase chain reactions (qRT-PCRs) were performed with TOPreal™ qPCR 2x PreMIX (TOP, Enzynomics, Korea) in a CFX96 Real-Time PCR Detection System (Bio-Rad, Hercules, CA). The qRT-PCR conditions were as follows: 4 min at 50°C and 10 min at 95°C, followed by 40 cycles of 10 s at 95°C, 15 s at 60°C, and 15 s at 72°C. All reactions were performed in triplicate, and the mean value was calculated. The specificity of the amplification was verified through the analysis of a melting curve generated by gradually heating the sample from 65°C to 95°C. The *α*-tubulin (TUA) was used as an internal control [38]. *C*_*T*_ values of qRT-PCR were obtained using CFX96 real-time controlling software (Bio-Rad, Hercules, CA). The fold change relative to control was calculated according to the method of Pfaffl [39].

In addition, the data of gene expression were analyzed using one-way analysis of variance (ANOVA), followed by the Student-Newman-Keuls multiple comparisons test for comparing the relative mRNA expression levels. Data are represented as mean ± SD, and *P* < 0.05 was considered statistically significant.

## 3. Results and Discussion

### 3.1. *CpCYP* cDNA and Genomics Characteristics

**Figure 1:**
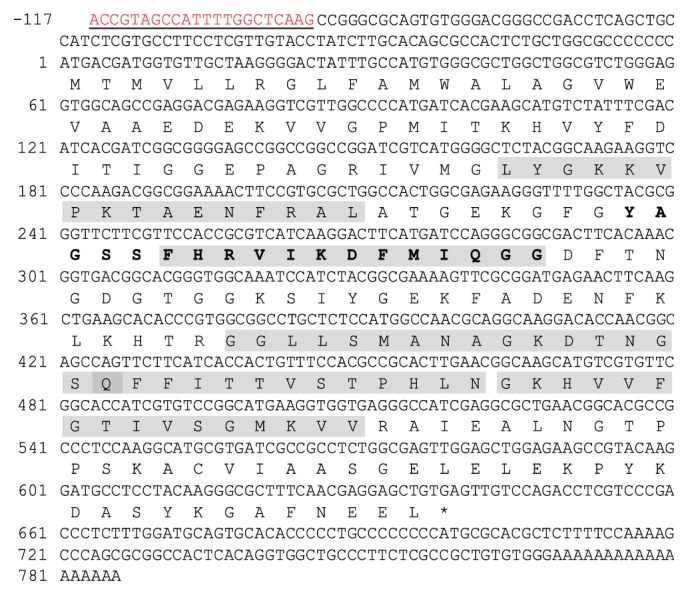
Nucleotide and amino acid sequences of CpCYP: DinoSL sequence in red and underlined; five signature motifs of* CpCYP* are highlighted (55–70, 84–96, 127–142, 142–154, and 155–170 aa). Sequence in bold: conserved site for cyclophilin-type peptidyl-prolyl* cis-trans* isomerase.

The cDNA sequence of* CpCYP* (GenBank number ABX0001) was 903 bp in length (Figure 1), coding 212 amino acids (aa) with a molecular mass of 22.72 kDa, and a theoretical isoelectric point of 8.53.

**Figure 2:**
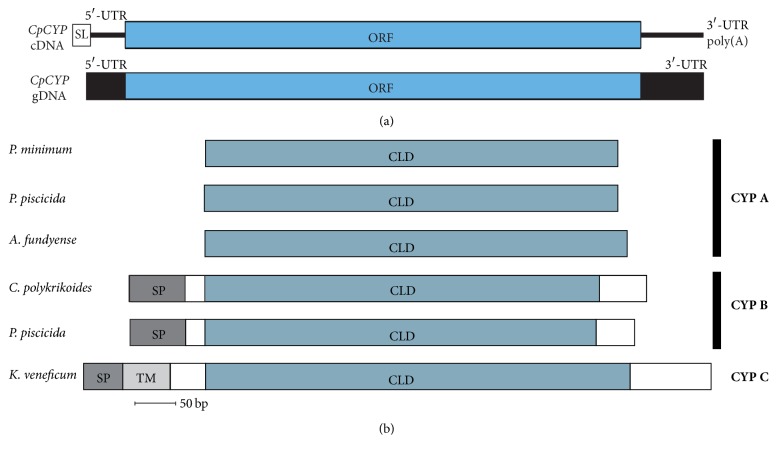
Comparison of cDNA and genomic DNA of* CpCYP* (a) and its predicted ORF primary structure with other dinoflagellates' CYPs (b). The proteins used here were taken from GenBank database, and their accession numbers are as follows:* Prorocentrum minimum* (AFD34244);* Pfiesteria piscicida* CYP A (ABI14282) and CYP B (ABI14285);* Alexandrium fundyense* (ABO47873);* Karlodinium veneficum* (ACU45296). SL: spliced leader; ORF: open read frame; SP: signal peptide; TM: transmembrane domain; CLD: cyclophilin-like domain.

Upon comparisons of CYP protein motifs, we found that the cyclophilin-type peptidyl-prolyl* cis-trans* isomerase proteins subfamily has a conserved pattern: [FY]-x(2)-[STCNLVA]-x-[FV]-H-[RH]-[LIVMNS]-[LIVM]-x(2)-F-[LIVM]-x-Q-[AGFT]-G (PROSITE accession number PS00170). Using motif search engines, a similar pattern (79 and 96 aa) was detected in our CpCYP (Figure 1). In addition, we identified a signal peptide of 23 aa located at the end of the N-terminus. Furthermore, a cyclophilin-like domain (CLD) was detected between 31 and 193 aa. Five signature motifs of the cyclophilin-type peptidyl-prolyl* cis-trans* isomerase were predicted as follows: 55–70, 84–96, 127–142, 142–154, and 155–170 aa. These patterns were matched with those of other dinoflagellates (Figure 2(b)); the structural differences of CYPs—presence of CLD alone or in combination with other domains (signal peptide, transmembrane domain, etc.)—are the key to determine their localization and therefore the group of CYPs they belong to. For example, PmCYP from* Prorocentrum minimum* showed a similar cyclophilins family conserved sequence and had five signature motifs; however, instead of a signal peptide, PmCYP had a cytoplasmic signal sequence predicting its cytoplasmic location [29].

As for the location of the gene coding for* CpCYP*, the presence of DinoSL sequence indicates that the gene is encoded in the nuclear genome of* C. polykrikoides* [9]. However, the presence of the N-terminal signal sequence in our* CpCYP* gene indicates that it is targeted to the ER protein secretory pathway [16, 19].

Genomic regions of* CpCYP* were amplified by PCR, and their sequences were compared to that of the cDNA. As a result, no intron was presented in the* CpCYP *coding genome (Figure 2(a)), which correlates with previous results showing that dinoflagellate genes contain very few or lack introns completely [40, 41]. In addition,* CYP* coding manner was investigated by long PCR using TaKaRa LA Taq kit, according to the manufacturer's instructions (the used primers are found in Table 1). However, we did not find any fragments, while this method could amplify intergenic spacer (IGS) regions of heat shock proteins (HSPs) from* C. polykrikoides* [42]. These results suggest that our CYP gene is present as a single copy and/or in different loci in chromosomes rather than a tandem arrangement.

Previously, 5 groups of cyclophilins were identified in yeast, according to their molecular mass and localization (CYP A, 17 kDa cytosolic protein; CYP B, 20 kDa secretory protein; CYP C, 18 kDa mitochondrial protein; CYP D, 22.8 kDa ER protein; and CYP 4, 33.4 kDa secretory protein) [43]. In mammals, the 5 groups have been suggested as well, comprising CYP A of 18 kDa soluble cytosolic proteins, CYP B of 22 kDa proteins with signal sequences for ER, CYP C of 23 kDa proteins with ER signal sequences, CYP D of 22 kDa proteins with putative mitochondrial signal sequences, and CYP 40 of 40 kDa proteins with low affinities to CsA [44]. In the dinoflagellates, to date, CYP protein has been isolated in 4 other species (*Alexandrium fundyense, Karlodinium veneficum, Pfiesteria piscicida*, and* Prorocentrum minimum*), with no further characterization; herein, we analyzed those sequences and identified 3 groups as follows: CYP A, CYP B to which our* CpCYP* belongs, and CYP C (Figure 2(b)). In* P. piscicida, *two groups of CYP have been recognized, suggesting the presence of more than one group in a dinoflagellate species; however, the presence of more CYP groups and their possible interactions are not to be found at present.

### 3.2. *CpCYP* Phylogenetic Relatedness to Other Eukaryotes

**Figure 3:**
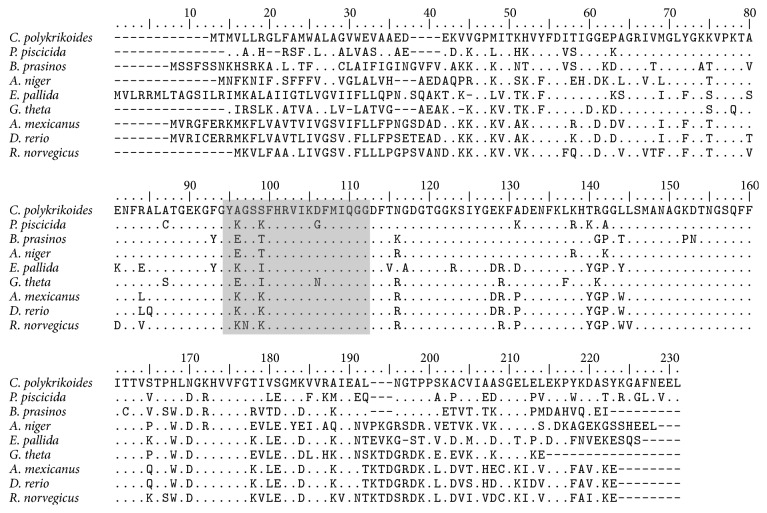
CpCYP amino acid sequence comparison with CYP B proteins of 8 eukaryotic species; a period (.) represents an aa identical to that of CpCYP, and a dash (–) marks an alignment gap; the cyclophilin-type peptidyl-prolyl* cis-trans* isomerase conserved site is highlighted in grey box. GenBank numbers of aligned proteins here are as follows:* Pfiesteria piscicida* ABI14285,* Bathycoccus prasinos* XP_007510149,* Aspergillus niger* XP_001401570,* Exaiptasia pallida* KXJ25954,* Guillardia theta* XP_005829570,* Astyanax mexicanus *XP_007235156,* Danio rerio* NP_998184, and* Rattus norvegicus* AAC25590.

The basic local alignment search tool (BLAST) searches using our CpCYP protein yielded 159 hits which all belonged to eukaryotic organisms. The search also showed that* CpCYP* has 76% identity with CYP of the dinoflagellate* Pfiesteria piscicida* (ABI14285) and 70% and 68% identities with the green algae* Micromonas commod*a (XP_002508058) and* Bathycoccus prasinos* (XP_007510149), respectively (Figure 3).

**Figure 4:**
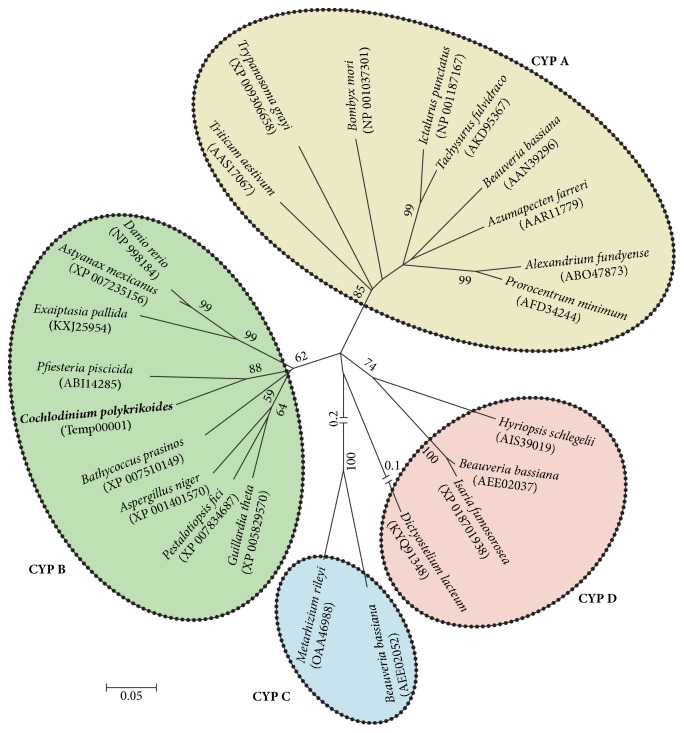
Phylogenic tree of CpCYP with other eukaryotic CYP proteins. The analysis was performed with MEGA5 (bootstrap method with 1,000 replicates), and the space bar represents the number of amino acid substitutions per site.* C. polykrikoides* is marked in bold.

In addition, a neighbor-joining (NJ) tree of CYPs displayed eukaryotic origin of CpCYP and divided four major clades as follows: CYP A clade (including dinoflagellates* Prorocentrum minimum* and* Alexandrium fundyense* and bivalve* Azumapecten farreri*), CYP B clade (including chlorophyte* Bathycoccus prasinos* and dinoflagellate* Pfiesteria piscicida*), CYP C clade (fungi* Beauveria bassiana *and* Metarhizium rileyi*), and CYP D (including fungus* Beauveria bassiana *and bivalve* Hyriopsis schlegelii*) (Figure 4). Similar results were obtained in the phylogenetic analysis of the red algae* Griffithsia japonica*, where CYPs from different species that belonged to the same CYP group were more closely related to each other than to CYPs from the same species in different groups [20]. These results confirm that the CpCYP identified in the present study (see Figure 2) belongs to cyclophilin B group, while our previous CYP identified from* P. minimum* should belong to CYP A [29]. Upon comparisons and BLAST searches, we only detected three groups (CYP A, CYP B, and CYP C) of CYPs from dinoflagellates (Figure 4), which were matched to the above results (Figure 3).

### 3.3. Effects of Environmental Stressors and Photosynthetic Efficiency

**Figure 5:**
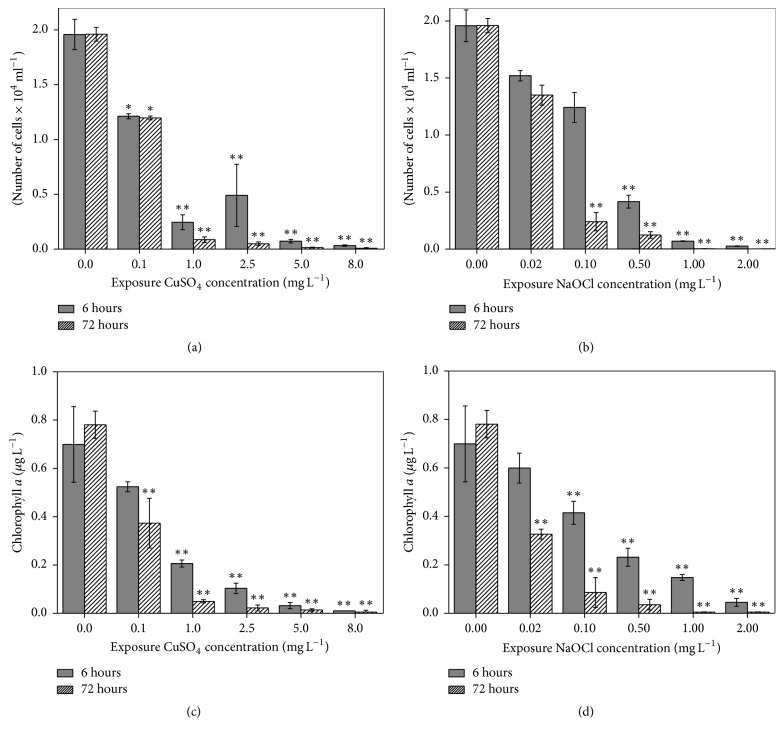
Variation in the number of cells (a, b) and chlorophyll *a* level (c, d) of* C. polykrikoides* after 6 and 72 h of exposure to biocides CuSO_4_ and NaOCl. Significant differences between the control and treated sample, as determined by one-way ANOVA, are highlighted. ^*∗*^*P* < 0.05; ^*∗∗*^*P* < 0.01.

Prior to* CYP* gene response of* C. polykrikoides* to typical contaminants, we assessed the effect of different doses of biocides CuSO_4_ and NaOCl over different exposure times, using some physiological parameters, including cell count, chlorophyll *a* levels, and photosynthetic efficiency. Cell count exhibited similar decreasing patterns in* C. polykrikoides* after 6 and 72 h exposure to CuSO_4_ and NaOCl (Figures 5(a) and 5(b)). After 6 h treatment, cell counts showed a significant decrease at the relatively higher concentrations (*P* < 0.05). Furthermore, after 72 h treatment, a significant reduction was observed, with more than 90% reduction. Such growth retardation effect is similar to those examined from the chlorophytes* Chlorella vulgaris* [45] and* Closterium ehrenbergii* [46] and the dinoflagellate* P. minimum* [47].

In addition to this, chlorophyll *a* levels showed a similar trend to cell count (Figures 5(c) and 5(d)). Similar to the present study, several reports [45, 46, 48] showed the inhibition of chlorophyll* a* content of algae by environmental contaminants. For example, Dia et al. [49] reported a dose- and time-dependent decrease in chlorophyll *a* levels of the blue-green algae* Microcystis aeruginosa *when exposed to CuSO_4_.

**Figure 6:**
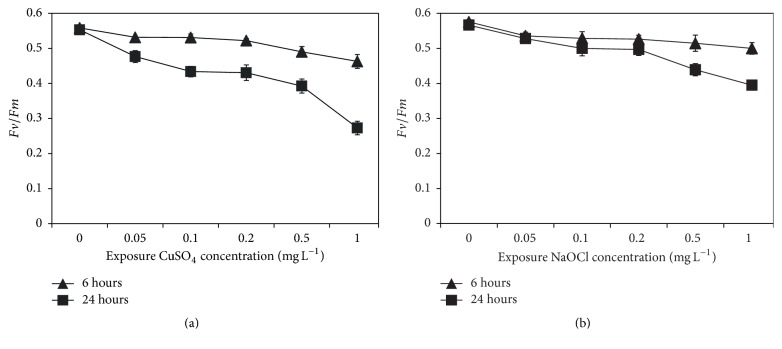
Variation in* Fv/Fm* ratio of* C. polykrikoides* after 6 and 24 h of exposure to different doses of biocides CuSO_4_ (a) and NaOCl (b).

As for photosynthetic efficiency (*Fv/Fm*), the results showed a dose-dependent reduction in response to CuSO_4_ and NaOCl. At 6 h exposure,* Fv/Fm* was slightly decreased with increased CuSO_4_ and NaOCl concentration. However, after 24 h incubation, the cells exposed to CuSO_4_ showed a much more significant decrease than those exposed to NaOCl (~0.3 and 0.4 at 1.0 mg L^−1^ for CuSO_4_ and NaOCl, resp.) (Figure 6). Our findings on inhibition of photosynthetic efficiency by environmental stress are in accordance with those observed by Guo et al. [38, 50] and Rocchetta and Küpper [51].

### 3.4. Effect of Toxic Chemicals on Transcription of* CpCYP*

**Figure 7:**
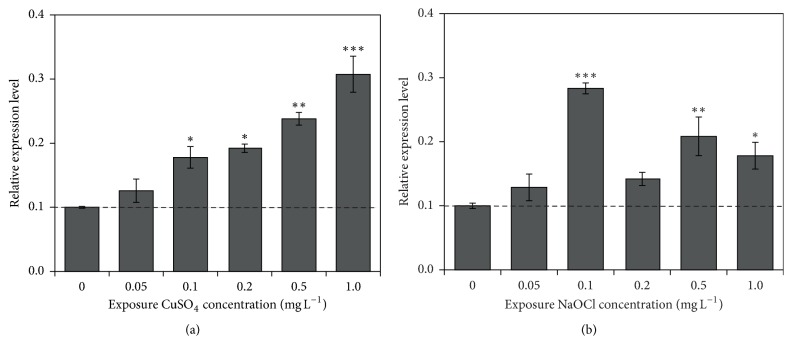
Effect of biocide pollutants CuSO_4_ (a) and NaOCl (b) on the expression of* CpCYP*. Significant differences between the control and treated sample, as determined by one-way ANOVA, are highlighted. ^*∗*^*P* < 0.05; ^*∗∗*^*P* < 0.01; ^*∗∗∗*^*P* < 0.001.

In this study, the transcriptional expression of* CpCYP *showed a different expression pattern after exposure to CuSO_4_ and NaOCl. In the case of exposure to CuSO_4_, the transcriptional expression level of* CpCYP* was significantly upregulated (*P* < 0.01), depending on the doses, showing 2.5- and 3.4-fold changes at 0.5 and 1.0 mg L^−1^ CuSO_4_, respectively, compared to that of the control. However, after NaOCl exposure, the transcriptional level was first increased, with 2.9- and 2.1-fold changes under 0.1 and 0.5 mg L^−1^ NaOCl treatments, respectively, and then decreased at relatively higher concentrations (Figure 7). Along with these results, it is clearly revealed that* CpCYP* gene expression is induced by biocide pollutants CuSO_4_ and NaOCl, but the expression patterns may depend on the dose and nature of toxicant. Our results on transcript abundance of CYP are in accordance with those of previous reports [52, 53]. For example, Wu and Lee [54] reported that the* CYP* transcription showed changes after exposure to excess copper in the marine macroalgae* Ulva fasciata*. In addition, Ponmani et al. [29] indicated that the gene expression of* CYP* in* Prorocentrum minimum* was significantly induced upon exposure to common pollutants (copper, sodium nitroprusside, and polychlorinated biphenyl). Taken together, upregulation of* CpCYP* in* C. polykrikoides* indicated its important role in stress-defense responses rather than previously known housekeeping functions.

### 3.5. Implications of the Dinoflagellate CYP

Dinoflagellates live in diverse habitats and seasons, and thus they are subjected to varied stressful conditions (e.g., water temperature changes, UV, and sudden exposure to toxic contaminants). These environmental stressors may be responsible for oxidative stress in cells [55], and in that case antioxidant proteins intervene as part of the cell's survival strategy. In the dinoflagellate* C. polykrikoides*, specific antioxidant genes and/or proteins, such as superoxide dismutase (SOD) and glutathione reductase (GR), have been detected by using large-scale transcriptome analysis of dinoflagellates [38]. In addition, we have discovered certain antioxidant genes, including catalase-peroxidase gene* (KatG)*,* GST*, and HSPs, which were suggested to be involved in the response of the cells protection against environmental toxicants [42, 55, 56]. The previous and present studies indicated that* CpCYP* might play a crucial role in combating environmental stress and facilitating molecular adaptation.

In addition, considering the expression level change of* CpCYP* by various environmental pollutants (such as heavy metals and biocide chlorine), it could be used as an early and rapid warning biomarker in ecotoxicity assessments [29].

In conclusion, this study determined the full-length sequence of cyclophilin from the harmful dinoflagellate* C. polykrikoides*. In addition, we characterized gene structure and phylogenetic affiliations to other CYPs and investigated the conserved motifs and signal peptides to determine the classification of cyclophilin type in dinoflagellates. Moreover, the transcriptional expressions of* CpCYP* were induced by biocide pollutants CuSO_4_ and NaOCl. These results combined shed light on the function of cyclophilins, to be more than a housekeeping gene, and the possibility of use as an ecotoxicity assessments biomarker. Further studies are necessary to identify different cyclophilin groups and the relationship between them and also reveal the effect of other toxicants on expressional responses in dinoflagellates.

## Figures and Tables

**Table 1:** The primers used in the present study.

Primer name	Nucleotide sequence (5′→3′)	Remarks
5-SL	CCGTAGCCATTTTGGCTCAAG	5′-RACE
CpCyp5R1	TGTCCTTGCCAGCATTGG	5′-RACE
CpCyp5R2	CGTCTTGGGCACCTTCTT	5′-RACE
B25	GACTCTAGACGACATCGA	3′-RACE
B26	GACTCTAGACGACATCGA(T)_18_	3′-RACE
CpCypF1	CATGACGATGGTGTTGCTAAG	3′-RACE/genomic DNA/full length
CpCypF2	GTTGCTAAGGGGACTATTTGC	3′-RACE/full length
CpCyp3F3	AAGAAGGTGCCCAAGACG	Full length
CpCypR1	GTTGAGCGTGCCCTTGTA	Genomic DNA/full length
CpCypR0	GACAACTCACAGCTCCTCGT	Genomic DNA/full length
CpCypR3	CACATGGCAAATAGTCCCCT	Intergenic DNA
CpCypF3	AGCCGTACAAGGATGCCT	Intergenic DNA
CpCypR4	CTTAGCAACACCATCGTCATG	Intergenic DNA
CpCypF4	GCTTTCAACGAGGAGCTGT	Intergenic DNA
CpCypR5	TTGATGACGCGGTGGAAC	RT-PCR
CpCypF5	TCTACGGCAAGAAGGTCC	RT-PCR
Cp-TUA2-F2	TTCTCGCGCATCGACCACAAG	RT-PCR
Cp-TUA2-R2	TCCATACCCTCGCCGACATAC	RT-PCR
